# Screening anthelmintic resistance to triclabendazole in *Fasciola hepatica* isolated from sheep by means of an egg hatch assay

**DOI:** 10.1186/s12917-015-0543-1

**Published:** 2015-08-28

**Authors:** David Robles-Pérez, José Manuel Martínez-Pérez, Francisco Antonio Rojo-Vázquez, María Martínez-Valladares

**Affiliations:** Faculty of Veterinary Medicine, University of León, Campus de Vegazana, 24071 León, Spain; Instituto de Ganadería de Montaña (CSIC-ULE), Finca de Marzanas, 24346 Grulleros, León, Spain

**Keywords:** *Fasciola hepatica*, Anthelmintic resistance, Egg hatch assay, Triclabendazole

## Abstract

**Background:**

In the present study, the detection of anthelmintic resistance to triclabendazole (TCBZ) in sheep infected by *Fasciola hepatica* was studied using an egg hatch assay (EHA). *F. hepatica* eggs were recovered from bile and faeces of infected animals by isolates with different grade of anthelmintic resistance to TCBZ: i) a resistant isolate (RT); ii) a susceptible isolate (ST); iii) naturally infected sheep by a susceptible field strain (FST). In the EHA the percentage of hatched eggs were calculated according to the following concentrations of TCBZ diluted in dimethyl-sulfoxide (DMSO): 0.05, 0.2, 1, 5, and 25 μg/ml.

**Results:**

In relation to the EHAs carried out with the eggs from bile of sheep infected by ST, differences were found in the percentage of hatched eggs between the control well, only with DMSO, and the two highest concentrations of TCBZ (5 and 25 μg/m) (*p* < 0.05). However, when we tested the drug with the eggs from the bile of sheep infected by RT, the percentage of hatched eggs was similar among all concentrations. Since the range of hatching varied between isolates, we calculated the ratio of the results of each concentration to its control value confirming the higher hatching in RT than in ST.

We developed an EHA with eggs recovered from faeces in order to avoid the slaughter of sheep. The results of the EHAs with the isolate ST showed differences in the percentage of hatching between the highest concentration (25 μg/ml) and the control well (*p* < 0.05); however, these differences were not confirmed under field conditions with the strain FST.

**Conclusions:**

The ovicidal effect of TCBZ in *F. hepatica* eggs from bile was shown using a commercial formulation diluted in DMSO with a minimum concentration of 5 μg/ml. However, in eggs recovered from faeces the results are not conclusive. The cleaning of eggs recovered from faeces is an important issue that should be reviewed and standardized before comparing results between susceptible and resistant isolates in this kind of EHA.

## Background

*Fasciola* spp. infects mammals worldwide, mainly ruminants, but also humans can become infected. In ruminants, and especially in sheep, the infection reduces feed conversion, growth, and meat and milk production. Moreover, it is one of the major causes of liver condemnations at abattoirs and interferes with fertility and fecundity [1].

The infection is usually caused, in temperate areas of the world, by the common liver fluke *F. hepatica*. Its prevalence is rising nowadays due to different factors such as climate change, man-made environmental modifications or the presence of anthelmintic resistance (AR) [1, 2]. AR is the result of repeated administration of the same anthelmintic; moreover, its development has been favoured by ineffective treatment due to the underdosing. AR is the result of repeated treatments of the same anthelmintic although its development is also favoured by the administration of underdosing or overdosing [3]. Currently, the most commonly used drug to control fasciolosis belongs to benzimidazole (BZ) family and is the triclabendazole (TCBZ). TCBZ has been the drug of choice for treating liver fluke infections in livestock for over 20 years [4] since it is the only anthelmintic effective against both *F. hepatica* stages, immature and mature flukes [5]. However, there are several reports describing resistant strains of *F. hepatica* to TCBZ all around the world, in Australia [6], Argentina [7] and also in different European countries [8–11]. Therefore, early detection of resistance is essential, since reversion to susceptibility does not seem to occur [12].

Some *in vivo* and *in vitro* tests have been developed to detect the AR in ruminants. Among the *in vivo* tests, the faecal egg count reduction test (FECRT) is based on the reduction of the number of eggs in faeces after the anthelmintic treatment [13]. Regarding *in vitro* tests, an egg hatch assay (EHA) has been described to detect BZ resistance in Trichostrongylidae [14, 15]. The EHA is based on the ovicidal properties of some BZs, and on the capacity of eggs from resistant isolates to embrionate and hatch at higher concentrations than those ones from a susceptible isolate [16]. Although the EHA was originally designed to detect AR in gastrointestinal nematodes (GIN), some studies have been carried out with *F. hepatica* eggs from gall bladder and/or faeces using TCBZ, albendazole (ABZ) and their sulphoxide metabolites [17–19].

The aim of this study has been to characterize the susceptibility and resistance of *F. hepatica* isolates to TCBZ by means of an EHA using eggs from gall bladder and faeces.

## Methods

### Isolates of *F. hepatica*

Eight experimentally infected sheep with two *F. hepatica* isolates having different levels of resistance or susceptibility to TBCZ were used. The susceptible isolate to TCBZ (ST) was the Shrewsbury/South Gloucester isolate (Ridgeway Research Ltd Company, UK); the TCBZ-susceptibility of this isolate was confirmed in a clinical trial by Martínez-Valladares et al. [11]. The resistant isolate to TCBZ (RT) was characterized by Álvarez-Sánchez et al. [10] in a flock located in the Spanish province of León; the egg reduction in this flock after the treatment of sheep with TCBZ was 81.8 % on 16 day after treatment and 75.7 % on 30 day after treatment. The molecular characterization of RT was recently described by Martínez-Valladares and Rojo-Vázquez [20].

On the other hand, naturally infected sheep with a TCBZ-susceptible field strain (FST) situated in Palencia, Spain, was also tested. The susceptibility of this strain was previously shown by Robles-Pérez et al. [21].

### Egg hatch assays

A commercial formulation of TCBZ (Fasinex®) diluted in dimethyl sulfoxide (DMSO) was used to carry out the EHAs. The concentration of TCBZ in this commercial formulation was 50 mg/ml. Dilutions of 10, 40, 200, 1000 and 5000 μg/ml were prepared to obtain a final concentration in the wells of 0.05, 0.2, 1, 5, and 25 μg/ml after adding 10 μl of each dilution to a total volume of 2 ml. In all EHAs, control wells with 10 μl of DMSO were included.

Eggs from faeces were obtained by sedimentation [22] from animals infected by ST and from a pool of faeces of sheep naturally infected by FST. Four sheep, two infected with ST and two with RT, were killed by injection of sodium pentobarbital (Dolethal®) into the jugular vein in order to recover eggs from the bile. *F. hepatica* eggs were directly recovered from the gall bladder and washed several times with tap water by sedimentation. The slaughter of animals complies with national regulations (R.D. 53/2013) and with all animal welfare standards, taking account all necessary moral and ethical issues in the use of experimental animals.

For the EHA, a 24 well cell culture plate was used and all anthelmintic concentrations were tested in duplicate. Into each well, 1890 μl of water, 100 μl of water with 30–50 eggs, and 10 μl of each dilution were placed. Two control wells containing 10 μl of DMSO, without TCBZ, were also included. Plates were incubated for 14 days at 25 °C in darkness. They were then placed under light for 2 h to stimulate hatching of the miracidia. The number of eggs hatched, embryonated, and unembryonated were counted. All EHA assays were repeated five times for each isolate.

### Data and statistical analysis

The percentage of hatched eggs was calculated for each isolate, using the following formula:

$$ \mathrm{Percentage}\ \mathrm{of}\ \mathrm{hatching} = \left(\mathrm{number}\ \mathrm{of}\ \mathrm{hatched}\ \mathrm{eggs}\ /\ \mathrm{total}\ \mathrm{number}\ \mathrm{of}\ \mathrm{eggs}\right) \times 100 $$

The number of eggs is the sum of hatched, embryonated, and unembryonated eggs (egg in morula stage, without miracidium). The results reported in this study are the mean of five repetitions of each EHA.

With the aim to compare two EHAs with different hatch ranges, a ratio of the results of each concentration to the control was calculated, using the following formula:

$$ \mathrm{Ratio} = \left(\%\ \mathrm{hatching}\ \mathrm{of}\ \mathrm{each}\ \mathrm{concentration}\ /\ \%\ \mathrm{hatching}\ \mathrm{of}\ \mathrm{control}\right) \times 100 $$

The data were analyzed using the statistical computer package for social sciences SPSS. A one-way ANOVA was used to assess differences. The Dunnett test was carried out to confirm significant differences between concentrations and the control group. Differences of less than 5 % were considered significant (*P* < 0.05).

## Results

### EHA with eggs from bile

**Fig. 1:**
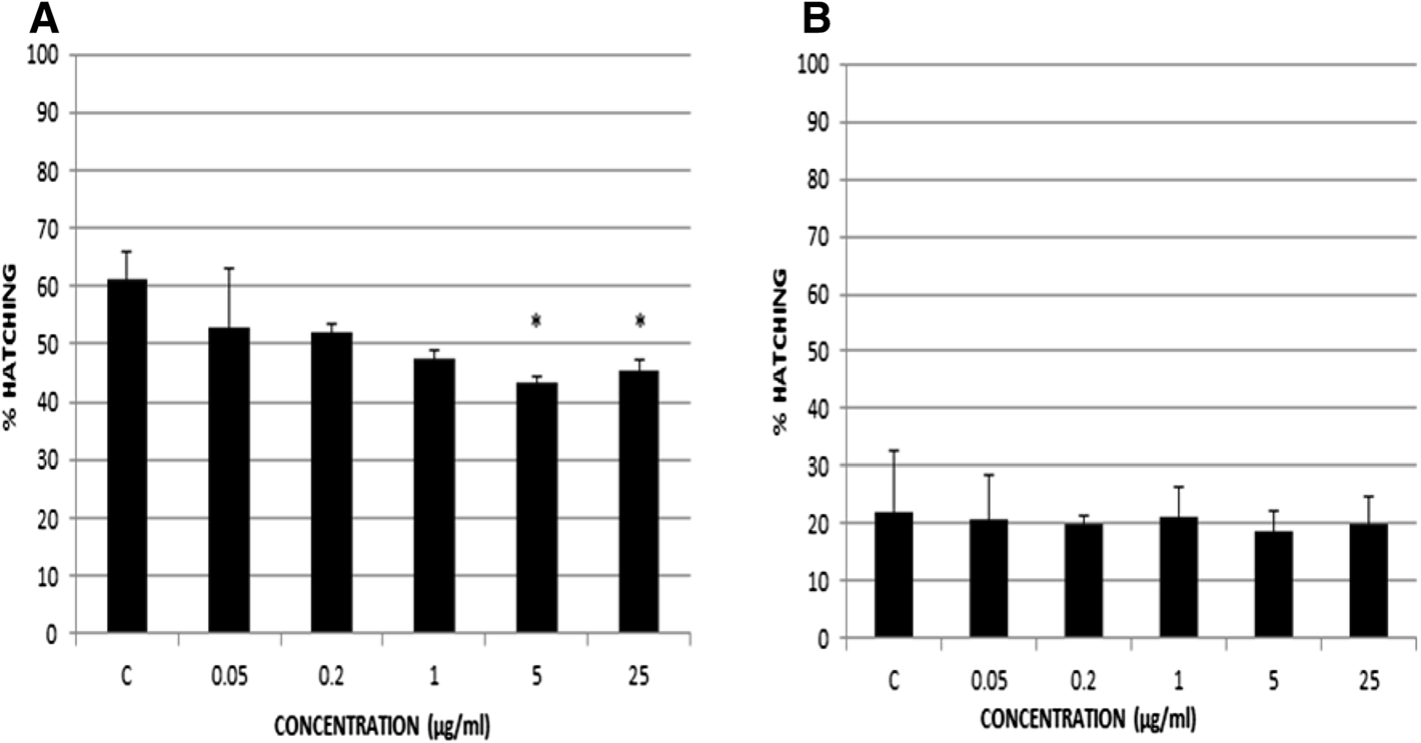
Percentages of hatched eggs in susceptible and resistant isolates using eggs from bile. The percentages of hatched eggs in the isolates ST (susceptible to TCBZ) (**a**) and RT (resistant to TCBZ) (**b**) were showed. Significant difference between concentration and control is indicated by *(*p* < 0.05)

In the EHAs carried out with the eggs from bile, we compared two isolates, one susceptible (ST) and another resistant (RT) to TCBZ (Fig. 1).

The percentages of hatching in ST isolate are shown in Fig. 1a. The results show that the lower the concentration of the drug is, the higher the percentage of hatched eggs, ranging from 43 to 53 %, with a value of 63 % in the control well. The differences between the two highest concentrations (5 and 25 μg/ml) and the control were significant (*p* < 0.05). On the other hand, the percentage of hatched eggs in RT ranged from 19 to 21 %, being 22 % in the control well (Fig. 1b). In this isolate, no significant differences were shown between concentrations.

**Fig. 2:**
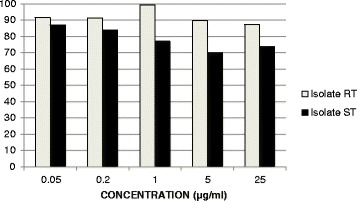
Ratio of hatched eggs from bile. The ratio for each concentration to its corresponding control for the susceptible and resistant isolates was showed

After calculating the ratio of the results of each concentration to its control value, in both isolates (Fig. 2), we confirmed the higher hatching in RT than in ST.

### EHA with eggs from faeces

**Fig. 3:**
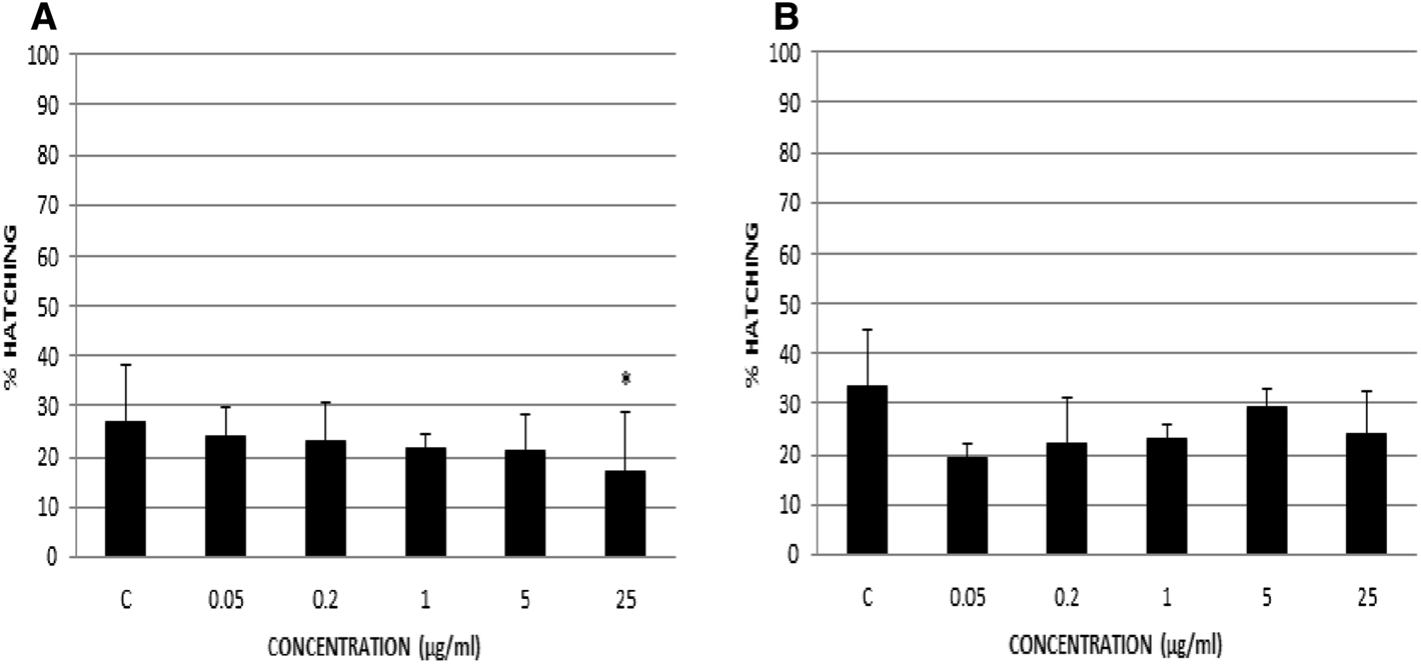
Percentage of hatched eggs in susceptible isolates using eggs from faeces. The percentages of hatched eggs in the isolates ST (susceptible to TCBZ) (**a**) and FST (field susceptible strain to TCBZ) (**b**) were showed. Significant difference between concentration and control is indicated by *(*p* < 0.05)

Figure 3 shows the results of percentage of hatched eggs in the susceptible isolates. The hatching percentage ranged from 17 to 23 %, with a control value of 26 % for ST. Significant differences were observed between the highest concentration (25 μg/ml) and the control well (*p* < 0.05) (Fig. 3a).

Using a field strain, FST, also susceptible to TCBZ (Fig. 3b), the percentages of hatching were similar between concentrations, ranging from 19 to 29 %, being 33 % in the control well; no significant differences were shown between concentrations.

## Discussion

The study of AR to BZs in ruminants infected by helminth parasites is an important issue to avoid its development and spread. Several authors have used the *in vitro* technique EHA in order to characterize strains of GIN and *F. hepatica* in sheep flocks [3, 14, 17–19, 23]. In the present study, the EHA has been adapted and modified to detect the AR to TCBZ in sheep infected by *F. hepatica*.

TCBZ metabolism includes ruminal and hepatic biotransformations in metabolites as TCBZ-sulphoxide, TCBZ-sulphone, hydroxy-TCBZ, hydroxy-TCBZSO and hydroxy-TCBZSO_2_ [24]. The TCBZ and its sulphoxide and sulphone metabolites contribute to anthelmintic activity and variations in the regional specificity in the levels of disruption to the tegument of the fluke are presents [25]. The drug and metabolites bind to β-tubulin which prevents the process of polymerization to form microtubules in the fluke [26]. Accordingly, the integrity of the surface membrane of the parasite is altered leading to damage to the integument and causing the death of the fluke [4].

Firstly, we carried out an EHA using a commercial formulation, Fasinex®, and eggs from bile. Both, TCBZ and its sulfoxide metabolite were used by Álvarez et al. [17] in a preliminary study testing two formulations of TCBZ in eggs from bile, one diluted with methanol and another one with DMSO. These authors showed a range of hatching of 45–80 % using concentrations of TCBZ between 5 and 20 nmol/ml, equivalent to 1.8–7.2 μg/ml; however, they did not find any ovicidal effect of TCBZ in egss from susceptible and resistant strains to this drug. Due to this fact, in the current study we extended the range of concentrations to test the efficacy of TCBZ in eggs from bile, between 0.05 and 25 μg/ml. After comparing the percentages of hatching between the susceptible (ST) and resistant (RT) isolates, we found only significant differences between the control and the two highest concentrations (5 and 25 μg/ml) in ST (*p* < 0.05), suggesting an ovicidal effect of the drug in this case. However, when the EHA was carried out with eggs from RT, no differences were found among concentrations (Fig. 1b). Recently, Fairweather et al. [18] tested a 60 μg/ml concentration of the sulfoxide metabolite of TCBZ in the same RT isolate that we used in the current study. This author found a very low level of hatching in RT (<2 %) and therefore they classified the isolate as susceptible. In the present study we showed a percentage of hatching of 20.5 % after testing the highest concentration (25 μg/ml); moreover, no significant differences were described between any concentration and the control using the same RT isolate. The reason of these different results in relation to resistance of RT could be due to the different formulations of TCBZ. Unlike our EHAs, Fairweather et al. [18] used a higher concentration and the sulphoxide metabolite of the drug, not the pure TCBZ.

With the aim to compare the results described between the susceptible and resistant isolates, we calculated a ratio for each isolate and concentration, since the hatching ranges of both isolates were different, probably due to the variability of the technique or the quality of eggs recovered (Fig. 2). The ratios show that the hatching was higher in RT, confirming its higher level of resistance.

On the other hand, with the purpose to avoid the slaughter of sheep to detect the AR using *in vitro* techniques, we developed an EHA with eggs of *F. hepatica* collected from faeces. With the aim to determine the repeatability of this technique using eggs from faeces, we only tested susceptible isolates, ST and FST. In this case, we observed that the hatching ranges were similar between each other (Fig. 3a and b), however, we only showed significant differences between the highest concentration (25 μg/ml) and the control well in ST. This finding was not confirmed in FST. It is important to note that the percentages of hatching in ST were lower than those obtained in the EHA using eggs from bile. The reason of the low hatching rates could be the presence of rest of faeces or impurities. Indeed, according to Rowcliffe and Ollerenshaw, [27] one of the critical factors for hatching is that the eggs must have become freed from the faeces. Robles-Pérez et al. [19] carried out EHAs to detect the resistance to ABZ using *F. hepatica* eggs recovered from faeces. In that case the percentages of hatching in the control wells were 33, 57 and 71 % for a susceptible strain and 49 % for a resistant strain. Therefore, there is a great variability in the hatching rates. It seems that the methodology to recover eggs from faeces is an important issue in the EHA, therefore, this step needs more review and standardization before comparing results between susceptible and resistant isolates.

## Conclusions

The ovicidal effect of TCBZ in *F. hepatica* eggs from bile was shown using a commercial formulation diluted in DMSO with a minimum concentration of 5 μg/ml. We compared the hatching rates of two isolates, one susceptible and another resistant to TCBZ, and we only found significant differences between the two highest concentrations (5 and 25 μg/ml) and the control well in the susceptible isolate. However, in eggs recovered from faeces the results are not conclusive. Significant differences were shown in the percentages of hatching between the highest concentration (25 μg/ml) and the control in a susceptible isolate, but these results were not confirmed under field conditions with another susceptible strain.

## Abbreviations

TCBZ: Triclabendazole
EHA: Egg hatch assay
RT: Resistant isolate
FST: Susceptible field strain
ST: Susceptible isolate
DMSO: Dimethyl-sulfoxide
AR: Anthelmintic resistance
FECRT: Faecal egg count reduction test
GIN: Gastrointestinal nematodes
ABZ: Albendazole
